# Drug Repurposing Strategy (DRS): Emerging Approach to Identify Potential Therapeutics for Treatment of Novel Coronavirus Infection

**DOI:** 10.3389/fmolb.2021.628144

**Published:** 2021-02-26

**Authors:** Biswa Mohan Sahoo, B. V. V. Ravi Kumar, J. Sruti, Manoj Kumar Mahapatra, Bimal K. Banik, Preetismita Borah

**Affiliations:** ^1^ Roland Institute of Pharmaceutical Sciences (Biju Patnaik University of Technology Nodal Centre of Research), Berhampur, India; ^2^ Kanak Manjari Institute of Pharmaceutical Sciences, Rourkela, India; ^3^ Department of Mathematics and Natural Sciences, College of Sciences and Human Studies, Prince Mohammad Bin Fahd University, Al Khobar, Saudi Arabia; ^4^ CSIR-Central Scientific Instruments Organization, Chandigarh, India

**Keywords:** drug, repurposing, strategy, therapeutic agents, treatment, coronavirus

## Abstract

Drug repurposing is also termed as drug repositioning or therapeutic switching. This method is applied to identify the novel therapeutic agents from the existing FDA approved clinically used drug molecules. It is considered as an efficient approach to develop drug candidates with new pharmacological activities or therapeutic properties. As the drug discovery is a costly, time-consuming, laborious, and highly risk process, the novel approach of drug repositioning is employed to increases the success rate of drug development. This strategy is more advantageous over traditional drug discovery process in terms of reducing duration of drug development, low-cost, highly efficient and minimum risk of failure. In addition to this, World health organization declared Coronavirus disease (COVID-19) as pandemic globally on February 11, 2020. Currently, there is an urgent need to develop suitable therapeutic agents for the prevention of the outbreak of COVID-19. So, various investigations were carried out to design novel drug molecules by utilizing different approaches of drug repurposing to identify drug substances for treatment of COVID-19, which can act as significant inhibitors against viral proteins. It has been reported that COVID-19 can infect human respiratory system by entering into the alveoli of lung via respiratory tract. So, the infection occurs due to specific interaction or binding of spike protein with angiotensin converting enzyme-2 (ACE-2) receptor. Hence, drug repurposing strategy is utilized to identify suitable drugs by virtual screening of drug libraries. This approach helps to determine the binding interaction of drug candidates with target protein of coronavirus by using computational tools such as molecular similarity and homology modeling etc. For predicting the drug-receptor interactions and binding affinity, molecular docking study and binding free energy calculations are also performed. The methodologies involved in drug repurposing can be categorized into three groups such as drug-oriented, target-oriented and disease or therapy-oriented depending on the information available related to quality and quantity of the physico-chemical, biological, pharmacological, toxicological and pharmacokinetic property of drug molecules. This review focuses on drug repurposing strategy applied for existing drugs including Remdesivir, Favipiravir, Ribavirin, Baraticinib, Tocilizumab, Chloroquine, Hydroxychloroquine, Prulifloxacin, Carfilzomib, Bictegravir, Nelfinavir, Tegobuvir and Glucocorticoids etc to determine their effectiveness toward the treatment of COVID-19.

## Introduction

Traditional methods of drug discovery is a complex, time-consuming, tedious, costly and risk process. There are various methods or steps involved in the drug discovery process which include random screening, molecular manipulation, computational strategy, drug metabolic study and serendipitous research (Myers and Baker, 2001). New drug candidates are developed mainly through different stages including identification of disease state or target, target validation, identification of lead, lead optimization, preclinical study, toxicity study, formulation, clinical trial, approval process, and marketing of the drugs with post marketing surveillance or safety monitoring etc (Hughes et al., 2011). As, these process are laborious, time-consuming and expensive with high risk of failure, drug repurposing is considered as novel approach to identify new therapeutic purposes of existing or already marketed or FDA approved drugs (Duarte, 2020). Drug repurposing is also represented as drug repositioning, drug reprofiling, drug rescuing, drug recycling, and therapeutic switching. Here, the drug repositioning involves the investigation of existing drugs for new therapeutic indications. For example the antiviral drugs like Remdesivir, Favipiravir and antimalarial drugs like Chloroquine, Hydroxychloroquine are repositioning for treatment of COVID-19. Similarly, the therapeutic switching is considered as a new strategy to enhance the RandD productivity by employing the new uses for stereoisomers and metabolites of existing or patented drugs. So, it is an effective approach for generating drug candidates with new therapeutic properties or pharmacological actions (Martorana et al., 2016).

On the other hand, COVID-19 infections become major public health issue of international concern since December 2019 (Hui et al., 2020). World health organization (WHO) announced the name for the novel corona virus disease as COVID-19 and declared as global pandemic on February 11, 2020 (Wang et al., 2020a). COVID-19 is an infectious disease caused by a newly discovered virus known as corona. This type of infectious is transmitted primarily through droplets produced when the infected person coughs, sneezes, or exhales. People with low immunity, old age, diabetes and other health problems associated with lungs, heart are more prone toward Covid-19 (Shereen et al., 2020). Currently, vaccines or therapeutic antibodies are not available to prevent the viral infection and more time is required to develop suitable therapeutic agents for acting against the pathogens (Li et al., 2020). So, there is urgent need to develop novel drugs for the prevention and treatment of COVID-19 infection effectively in a short span of time schedule. Thus, drug repurposing strategy is applied to obtain new drugs from existing old drugs with safety and efficacy to meet the need of current emergency for COVID-19 instead of applying the traditional drug development approaches (Firas et al., 2020; Hafeez et al., 2020).

### SARS-CoV-2: Genomic Structure and Pathogenesis

Corona virus disease was first discovered as acute respiratory infection in case of domestic chicken during 1930s. But, the human corona virus was discovered in United States and United Kingdom during in 1960s (Zhu, 2020). There are two types of corona viruses identified such as severe acute respiratory syndrome corona virus (SARS-CoV) and the Middle East respiratory syndrome corona virus (MERS-CoV). The novel coronavirus (nCoV) is mutated from the SARS and MERS. These are highly identical, pathogenic to humans and the strains are recognized as the SARS-CoV-2. The corona viruses cause severe and fatal respiratory tract infections in human beings (Cooke and Shapiro, 2003). Genetically, corona virus is divided into four groups such as α (group-1), β (group-2), γ (group-3) and δ (group-4). Currently, the entire world is under a threat of Covid-19 (*β*-coronavirus or group-2) infection which was emerged in Wuhan, China during December 2019. From genetic studies, it was found that bats are the primary sources and considered as hosts for the strains of viruses such as SARS-CoV and MERS-CoV before spreading to humans (Pirone et al., 2020).

**FIGURE 1:**
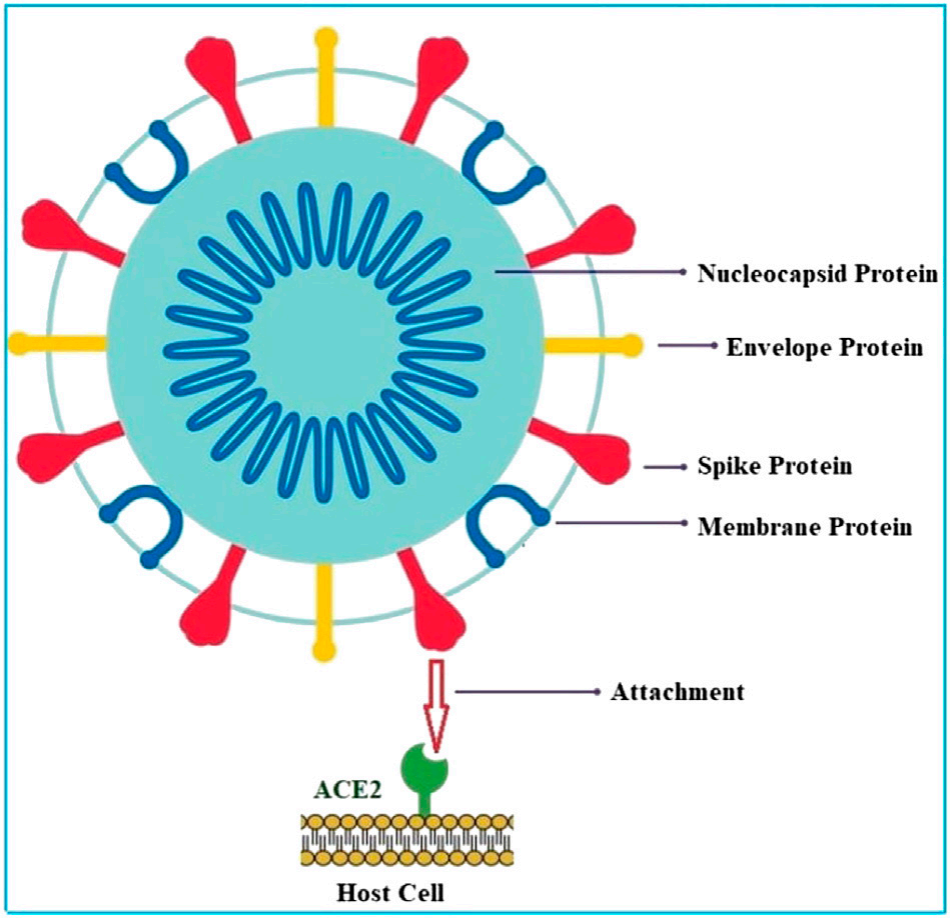
Structure of SARS-CoV-2 and target binding sites.

The structure of corona virus is observed as spherical or pleomorphic in shape. It contains single stranded RNA genomes in the size ranging from 26 to 32 kilo bases with a nucleoprotein within capsid consist of matrix protein (Ibrahim et al., 2020). There are four structural proteins present in corona virus such as spike (S), envelope (E), membrane (M), and nucleocapsid protein (N). The S, E, and M proteins are present within the virus whereas the N-protein is present within the nuclear membrane. The envelop bears club shaped glycoprotein projections called spike protein (Figure 1). This protein is a type-1 transmembrane protein which contains about 1,160 amino acids. The envelope has a crucial role in pathogenesis of viral infection as it promotes the assembly and release of virus. S-protein of corona virus plays key role for the induction of neutralizing-antibody and T-cell responses, as well as protective immunity during infection with corona virus. There are two subunits such as S1 and S2, present in S-protein. The S1 subunit contains a receptor-binding domain (RBD) which engages with ACE-2 of the host cell receptor and the S2 subunit mediates the fusion between host cell membranes and viral particles (Du et al., 2009; Chen et al., 2020). It has been reported that Covid-19 can infect human respiratory system through the mucous membranes of nasal and larynx mucosa and then enters into alveoli of the lungs via respiratory tract. The virus primarily attacks the organs such as the lungs, kidney, heart, and gastrointestinal tract (GIT) which express the angiotensin converting enzyme 2 (ACE2). The E and M proteins are mainly involved in viral assembly whereas the N-protein is essential for assembly of RNA genome (Prabakaran et al., 2004).

### Focused Treatments for COVID-19

**FIGURE 2:**
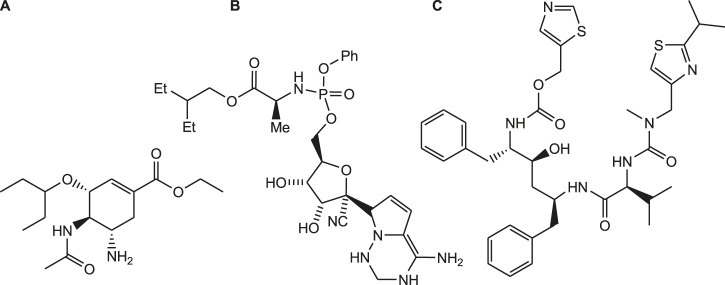
Structure of oseltamivir **(A)**, remdesivir **(B)** and ritonavir **(C)**.

The current pandemic situation of COVID-19 requires an urgent development of potential strategies to protect people who are suffering with high risk of corona virus infection (Guo et al., 2020). As the drug discovery and development process is lengthy and tedious process, research is going on for the rapid development of novel drug candidates. As per WHO recommendation, clinical trials are conducted to investigate the potential effect of anti-viral drugs such as remdesivir, favipiravir, oseltamivir and ritonavir on COVID-19 (Figure 2) (Wang et al., 2020b).

Similarly, anti-malarial drugs such as Chloroquine phosphate and Hydroxychloroquine sulphate were also used for the prevention and effective management of COVID-19 infection in emergency cases. But, these drugs are not used satisfactorily for the patients with conditions like diabetes, hypertension and cardiac problems (Vincent et al., 2005). Recently, anti-inflammatory corticosteroid drug like dexamethasone has been recommended to treat severe COVID-19 patients with cytokine release syndrome (CRS). But, glucocorticoids are the steroidal drugs that should not be used in patients infected with COVID-19 and pneumonia unless there are other indications like exacerbation of chronic obstructive pulmonary disease (COPD). Glucocorticoids are also associated with high risk for mortality in patients with influenza and delayed viral clearance in patients infected with MERS-CoV. Although, glucocorticoids are widely used for the treatment of SARS, there is no suitable evidence for its beneficial effects and any adverse effects (Lee et al., 2004).

Human monoclonal antibody based drug like sarilumab inhibits interleukin-6 (IL-6) receptor and is now being tested against COVID-19. Similarly, tocilizumab is also recombinant humanized monoclonal antibody and used for treatment of rheumatoid arthritis. It exhibits its action by inhibiting the function of IL-6 by binding specifically to the receptor. Currently, it is used in case of patients suffering from severe COVID-19 with elevated levels of IL-6 and cytokine storms. Leronlimab is a monoclonal antibody based drug and it is identified to bind with CCR5 receptor on the CD4^+^ T-lymphocytes. This drug is investigated under clinical trials for treatment of COVID-19 (Biggioggero et al., 2018).

**FIGURE 3:**
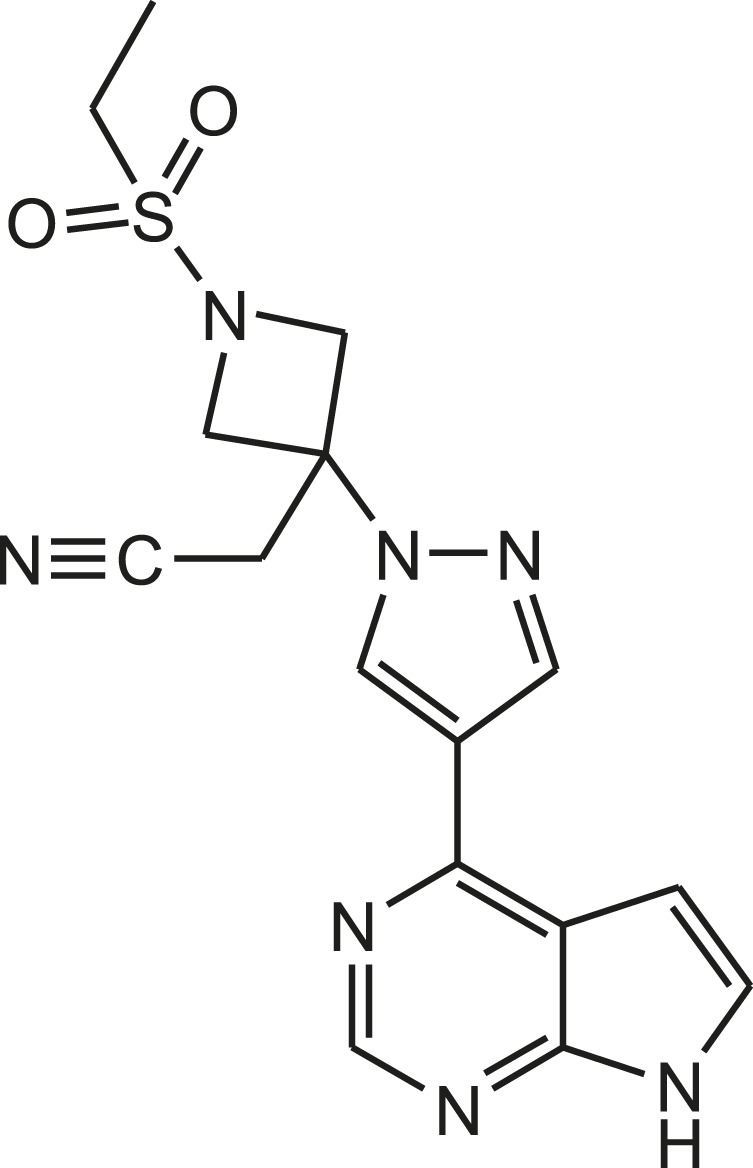
Structure of baricitinib.

Similarly, baricitinib is orally bioavailable selective and reversible inhibitor of Janus kinases-1 and 2 (JAK1/2), with potential anti-inflammatory, immunomodulating and antineoplastic activities (Figure 3). JAK kinases are intracellular enzymes involved in inflammation, immune function and signaling of cytokines. Baricitinib inhibits the activation of JAK1/2 that leads to the inhibition of the JAK-signal transducers and activators of transcription (STAT) signaling pathway. This decreases the production of inflammatory cytokines and may prevent the inflammatory response. In addition to this, baricitinib may stimulate apoptosis and diminish the proliferation of JAK1/2-expressing tumor cells. Based on this evidence, it is also used as potential agent for the treatment of 2019-nCoV acute respiratory disease. But, these drugs are assessed in clinical trial to establish their safety and efficacy (Richardson et al., 2020).

Due to the lack of availability of approved therapeutics for treatment of COVID-19, there is urgent need of the research group to obtain novel drug candidates with the ability to treat this disease. Hence, computational approach like drug repurposing is applied to identify new therapeutics within a short period of time to overcome the challenges of antiviral drug therapy for the effective treatment of COVID-19 (Hodos et al., 2016).

### Computational Tools Used for Drug Repurposing

The drug repurposing study is considered as an emerging strategy to discover new therapeutic indications of already approved or exiting drugs as this method is time-efficient and cost-effective (Peyvandipour et al., 2018). There are various databases and computational tools available for drug repurposing which include e-Drug3D, DrugPredict, Drug Bank, Promiscuous, Mantra2.0, PharmDB, DRAR-CPI, repoDB, Repurpose DB, DeSigN, Cmap, DPDR-CPI etc (Lee et al., 2012). These tools provide information regarding three dimensional structures of the compounds and target proteins as well as their binding interactions in the 3D space. Further, the targets can be categorized into two types such as virus-based and host-based targets based on their binding affinities. From, x-ray crystallography and NMR spectroscopic studies, structure of different targets of corona virus are identified that include spike protein, envelop protein, membrane protein, protease, nucleocapsid protein, hemagglutinin esterase and helicase etc (Prajapat et al., 2020).

The infection by SARS-CoV-2 initiates with the viral entry mediated through the interaction of the spike (S) protein with the host ACE2 receptor followed by cleavage of the S protein by the host transmembrane serine protease 2 (TMPRSS2) prior to the fusion to the host cell surface. The entry of Coronavirus is mainly achieved by binding of the virus to the ACE2 receptor of host in the cell surface via receptor mediated endocytosis pathway. So, the inhibition or modulation of ACE2 receptor is considered as one of the host-based strategy for the management of SARS-CoV-2 infection. Losartan and telmisartan are recognized as ACE2 receptor inhibitors and used clinically for the treatment of high blood pressure, heart failure and diabetic kidney disease (Supplementary Table S1).

**FIGURE 4:**
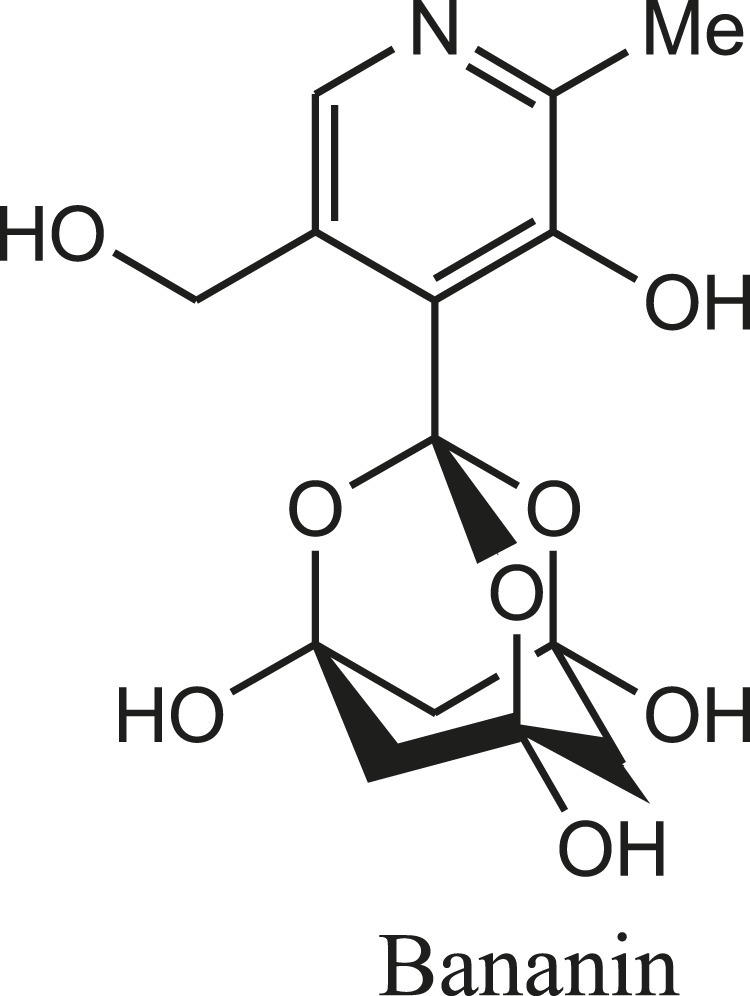
Structure of SARS-CoV helicase inhibitor.

Similarly, Bananins are adamantine derivatives (bananin, iodobananin, vanillinbananin and eubananin) and reported as potent inhibitors of the both helicase activities and replication of SARS coronavirus (Figure 4) (Carmen et al., 2020).

The e-Drug 3D data base provides the information regarding 3D structures of FDA approved drugs (Pihan et al., 2012). This information can be employed for virtual screening such as fragment-based drug design and drug repurposing. Drug Predict determines the interactions of drug molecules with binding sites of non-target proteins by using docking calculations. This data is useful for predicting new therapeutic effects of drug substances. Promiscuous database allows for getting complete information regarding drug-target interaction, protein-protein interaction and side effects of the drugs (Eichborn et al., 2011). It is proved to be beneficial for drug repurposing by correlating between structural similarity of drug molecules and their adverse effects to drug-receptor interactions. Moreover, the visualization tools of Promiscuous helps to explore and determine the binding interaction between drugs and targets, as well as identify the drug candidates for repurposing. Mantra 2.0 allows the researcher to provide profiles of gene expression before and after treatment of drugs in one or multiple cells (Carrella et al., 2014). This data helps to determine the mode of action of drugs which provide opportunities for drug repurposing. PharmDB is an integrated database which provides information regarding the drug development, disease states, associated proteins, and their binding interactions with drug molecules. Similarly, DrugBank (DB) provides detail information about drugs (i.e., physicochemical and pharmacological property) and target proteins (i.e., structure, sequence and pathway). Repurpose DB explores the details about disease conditions, side effects, and drug-targets interactions (Wishart et al., 2006; Lamb, 2007).

Currently, Excelra’s COVID-19 drug repurposing database has been developed by expert scientific teams to identify the safe and effective therapeutic options for the treatment of novel coronavirus disease (Supplementary Table S2). It is an open-access database and provides information regarding approved small molecules and biologics, which can rapidly enter into either phase 2 or 3 clinical trial, or may be directly used against COVID-19. In addition to this, it includes information about promising drug candidates which are in various phases of pre-clinical, clinical and experimental stages of drug discovery and development process (Campillos et al., 2008; Brown and Patel, 2017).

### Drug Repurposing as a Strategy to Identify New Therapeutic Agents

Drug repurposing is considered as an emerging strategy of computational approach to identify new therapeutic agents within a short period of time for effective treatment of COVID-19 (Xue et al., 2018). This approach is based on virtual screening of drug libraries to identify suitable drugs and their binding interactions with target protein by using computational tools such as molecular similarity and homology modeling etc (Sliwoski, 2013). Further, molecular docking and binding free energy calculations are also performed to determine the binding affinities and interactions among drug molecules and receptors (Sumudu and Leelananda, 2016).

**FIGURE 5:**
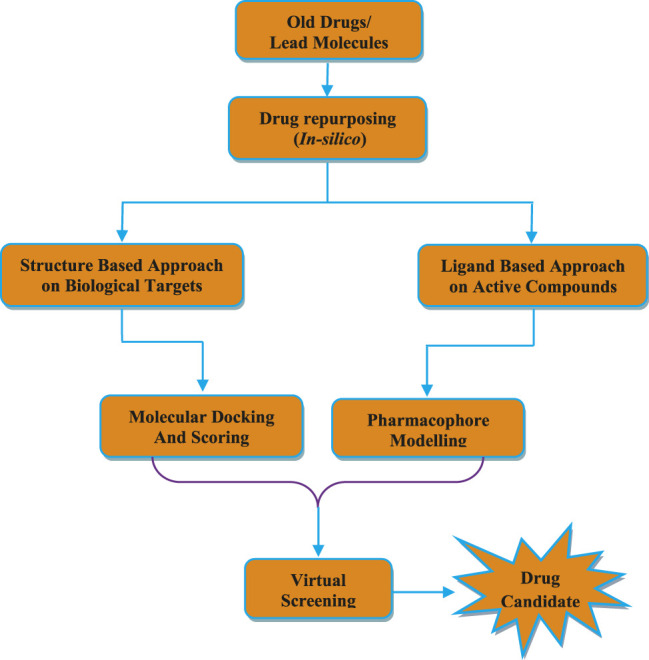
Various steps involved in drug repurposing study.

Drug repurposing is also called as drug repositioning or drug reprofiling or drug rescuing. In this process, new medicinally active agents are designed from the old or existing or pro-drugs or FDA approved clinically used drugs (Figure 5) (Deotarse et al., 2015). Recently, *in silico* methods are employed along with the utilization of structure based drug design (SBDD), ligand based drug design (LBDD) and artificial intelligence (AI) technology to accelerate the drug repurposing process (Ashburn and Thor, 2004). Drug repurposing provides several advantages such as reduction of the time period spent during research, reduction in complexity and cost of process in comparison with traditional approaches of drug discovery process (Chong and Sullivan, 2007). It is estimated that 10–12 years are required for the development of a new drug molecules in traditional drug discovery approach. While, the estimated time is between 1 and 3 years in case of drug repositioning method. The average expenditure required to obtain a new pharmacologically active drug to market is USD 1.24 billion by traditional drug development process. Whereas, in case of drug repurposing process, it costs around ≤60% expenditure of traditional drug discovery methods (Napolitano et al., 2013). Due to the availability of previously collected data related to structural optimization, pharmacokinetic, toxicological, clinical efficacy and safety profile of drugs during traditional drug discovery approach, there is reduction in time of drug development with lower cost and reduced risks of failure or high success rate in drug repurposing (Wu et al., 2013). Traditional methods of drug discovery process mainly focus on development of drugs to treat chronic and complex diseases, whereas drug repositioning approach primarily focus on the development of drugs for emerging infectious diseases which are difficult to treat and neglected diseases (Li, 2015).

**FIGURE 6:**
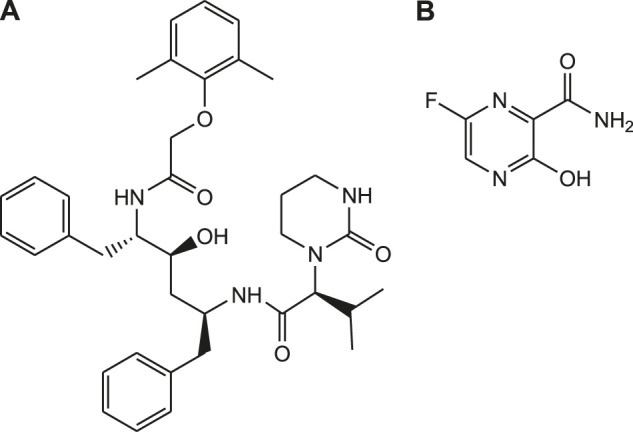
Structures of lopinavir **(A)**, favipiravir **(B)**.

Antiviral drugs such as favipiravir, remdesivir, lopinavir are previously used clinically for the treatment of SARS, MERS and AIDS (Figure 6). Currently, drug repurposing study is performed to investigate effectiveness of these drugs against COVID-19 (Walmsley et al., 2002). Remdesivir is a novel nucleotide analogue that inhibits viral RNA polymerases and used as broad-spectrum antiviral drug (Eastman, 2020). Similarly, favipiravir (T-705) is a synthetic prodrug with antiviral activity. It is developed by structural modification of the pyrazine moiety of T-1105. It is active against the influenza virus infections by inhibiting the influenza viral RNA-dependent RNA polymerase (RdRp) enzyme. Based on this mechanism of action, clinical studies have been conducted to assess the efficacy of favipiravir in the management of COVID-19 (Agrawal et al., 2020; Katakam et al., 2020).

Lopinavir is protease inhibitor and used as an antiretroviral therapy for the treatment of HIV infections. So, the drug repurposing approach provides an insight about the therapeutic activity of these drugs to treat COVID-19. It was observed that HIV-protease inhibitors and RNA-dependent RNA polymerase inhibitors exhibited promising aspects of binding interaction toward target proteins of SARS-CoV-2 (Joo et al., 2012).

**FIGURE 7:**
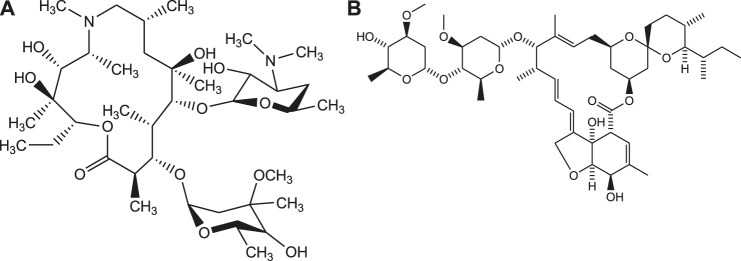
Structures of azithromycin **(A)** and Ivermectin **(B)**.

Similarly, the anti-malarial drugs such as Chloroquine and Hydroxychloroquine possess immune-modulating effect on humanbeings and found to be effective against viral infection at early stages of COVID-19 (Jeon, 2020). Further, some of the antibiotics such as azithromycin, teicoplanin, oritavancin, dalbavancin are repurposed computationally to identify their effectiveness for treatment of Covid-19. Recently, the antiparasitic drug like Ivermectin is also repurposed to find out its therapeutic potential for the effective management of COVID-19 (Figure 7) (Ciemny, 2018; Kumar, 2020).

Currently, extensive investigations are carried out on various existing drugs and subjected for clinical trials to identify the effective therapeutic agents against infection of COVID-19. From these observations, it was found that some of the trials were found to be completed, whereas some drugs are in the recruiting stage and found to be active. The first randomized clinical trial (NCT04261517) of hydroxychloroquine on COVID-19 patients exhibited the drug to be effective against Corona virus infection. The swab sample of these patients was found to be negative, but the clinical trial lacked a larger sample size (Beigel et al., 2020).

Recently, Grein et al., 2020 reported that there was clinical improvement of 68% of the patients after administration of antiviral drug like remdesivir. It was further reported that there is reduction of viral load in mice treated with remdesivir by *in-vivo* study on MERS-CoV mice model. Similarly, Ribavirin, a broad-spectrum antiviral drug when treated in combination with lopinavir or ritonavir reduced the risk of ARDS and death in SARS patients. From the clinical trial on favipiravir, it was found that it is therapeutically more active as compared to lopinavir and ritonavir (Dong et al., 2020).

Zhavoronkov, 2018 reported the drug repurposing study of various drugs such as Prulifloxacin, tegobuvir, bictegravir and nelfinavir for the management of COVID-19. These drugs are selected by utilizing high-throughput computational screening method. From the results, it was observed that these drugs possess high binding affinity toward SARS-CoV main protease. Prulifloxacin is reported as prodrug which is metabolized rapidly into ulifloxacin *in vivo.* But, it can be used as a lead compound for any structural modification and optimization to design more effective inhibitors of main protease of COVID-19 (Matera, 2006). Tegobuvir is a novel non-nucleoside inhibitor (NNI) of HCV RNA replication with significant antiviral activity in patients with genotype-1 chronic HCV infection. Further, both bictegravir and nelfinavir are used as anti-HIV drugs. Bictegravir is a potent HIV-1 integrase inhibitor, which is able to prevent the HIV infection efficiently (Davis et al., 2012). Whereas, nelfinavir is a protease inhibitor that inhibits the cleavage of the polyprotein gag-pol. It is reported that these drug molecules have the capacity to block the active sites or hinder the dimer formation of viral protein. Therefore, these drugs serve as promising therapeutic agents for drug repurpose to act against 2019-nCoV (Tsiang et al., 2016).

Similarly, Zhang et al., 2020 performed the therapeutic drug targeting of main protease (Mpro) of COVID-19 by high throughput screening. The main protease of SARS-CoV is crucial for the life cycle of virus and this protease displayed 96.1% of similarity with the main protease of COVID-19. So, the main protease is considered as suitable target for development of novel drugs to treat the corona virus infection. The 3D structures and sequences of SARS-CoV main protease were obtained from protein data bank. The crystal structure of main protease monomer with PDB ID: 5n5o was considered as target protein. The receptor was prepared by removing water molecules, adding hydrogen atoms and computing charges by using AutoDock tools. The 3D structure of drug molecule was displayed by PyMOL v2.3. From the docking study, it was revealed that the binding energies of Prulifloxacin were found to be −8.2 kcal/mol, −8.2 kcal/mol, −7.9 kcal/mol respectively at three binding sites (cave adjacent to N-terminal, the dimer joint groove and its back side) of viral main protease. Whereas, the binding energy between bictegravir and protease was found to be 7.3 kcal/mol and −8.3 kcal/mol at the active site and the joint groove respectively. Similarly, the binding energy associated with nelfinavir, tegobuvir and protease was found to be −8.6 kcal/mol and −8.9 kcal/mol respectively (Supplementary Table S3). These observations suggested that these drug molecules have ability to block the active sites of the viral protein. Hence, these drugs may be used as suitable agents for repurpose against COVID-19.

**FIGURE 8:**
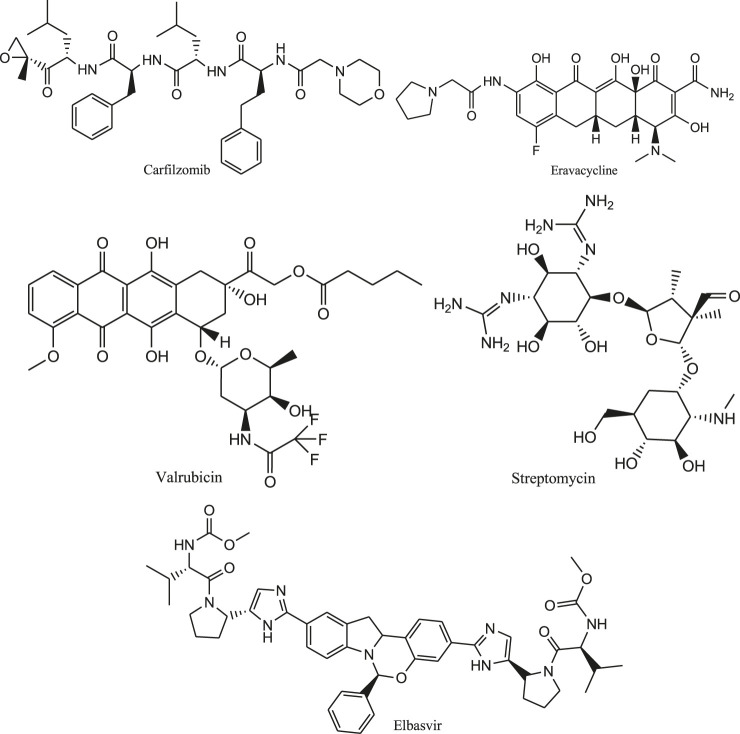
Structures of inhibitors of SARS-Cov-2 main protease.

Wang. (2020) performed the computational drug repurposing study to determine suitable drug molecules for effective treatment of COVID-19. So, virtual screening is carried out for both drugs under clinical trials and drugs approved by FDA. For this purpose, molecular dynamics simulation study was performed followed by calculation of binding free energy by using an endpoint method called MM-PBSA-WSAS (Molecular Mechanics-Poisson Boltzmann Surface Area-Weighted Solvent-Accessible Surface Area). Glide program is used for the docking study. The potential inhibitors of SARS-Cov-2 main protease such as Carfilzomib, Eravacycline, Valrubicin, lopinavir, elbasvir and Streptomycin (Figure 8) are considered for this repurposing study.

It was found that the structure of SARS-Cov-2 main protease is mostly similar to SARS-Cov protease (PDB Code 3TNT46) and less similar to MERS-Cov protease (PDB Code: 5WKK47). Carfilzomib (DB08889) is an approved anti-cancer drug which is acting as a proteasome inhibitor. It possesses the MM-PBSA-WSAS binding free energy of −13.8 kcal/mol. Similarly, the neutral form of Streptomycin (DB01082) has a MM-PBSA-WSAS binding free energy of −7.92 kcal/mol which is much better than the charged form (−3.82 kcal/mol) as presented in Supplementary Table S4. The difference is due to different electrostatic properties between the neutral and charged molecules. The result of this experiment provides information to accelerate the rational drug design for targeting SARS Cov-2 main protease.

**FIGURE 9:**
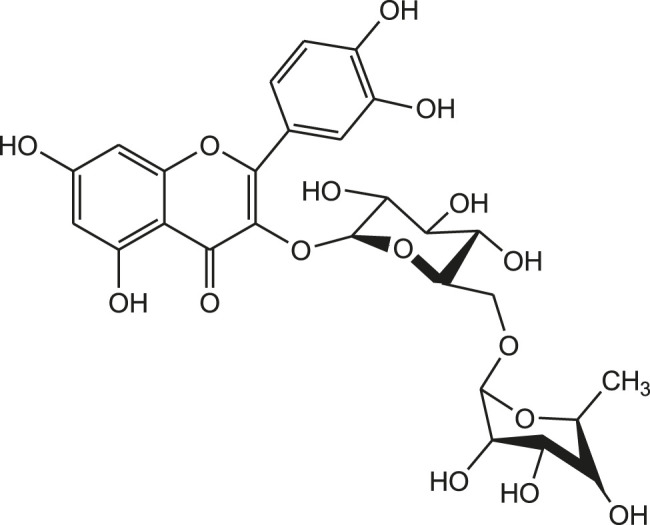
Chemical structure of Rutin (Docking score: 9.16 kcal/mol).

Recently, the crystal structure of 3-chymotrypsin-like proteinase (3-CLpro) of COVID-19 is resolved and the proteinase is identified as a promising drug target for novel coronavirus. The 3D structure of COVID-19 3CLpro complex can be obtained from PDB with ID: 6LU7. It is demonstrated that chymotrypsin-like protease is vital in the viral life cycle and the protease is found to be stable inside the coronavirus. Hence, the COVID-19 3CLpro is considered as a potential target for the development of new anti-COVID-19 drug molecules. Based on the prediction of machine learning (ML) and molecular docking study, flavonoid like Rutin is found as potential inhibitor of COVID-19 3CL proteinase. From the result analysis, it was observed that the compound Rutin exhibit docking score of -9.16 kcal/mol and AUC: 0.990 (Figure 9) (Xu et al., 2020).

**FIGURE 10:**
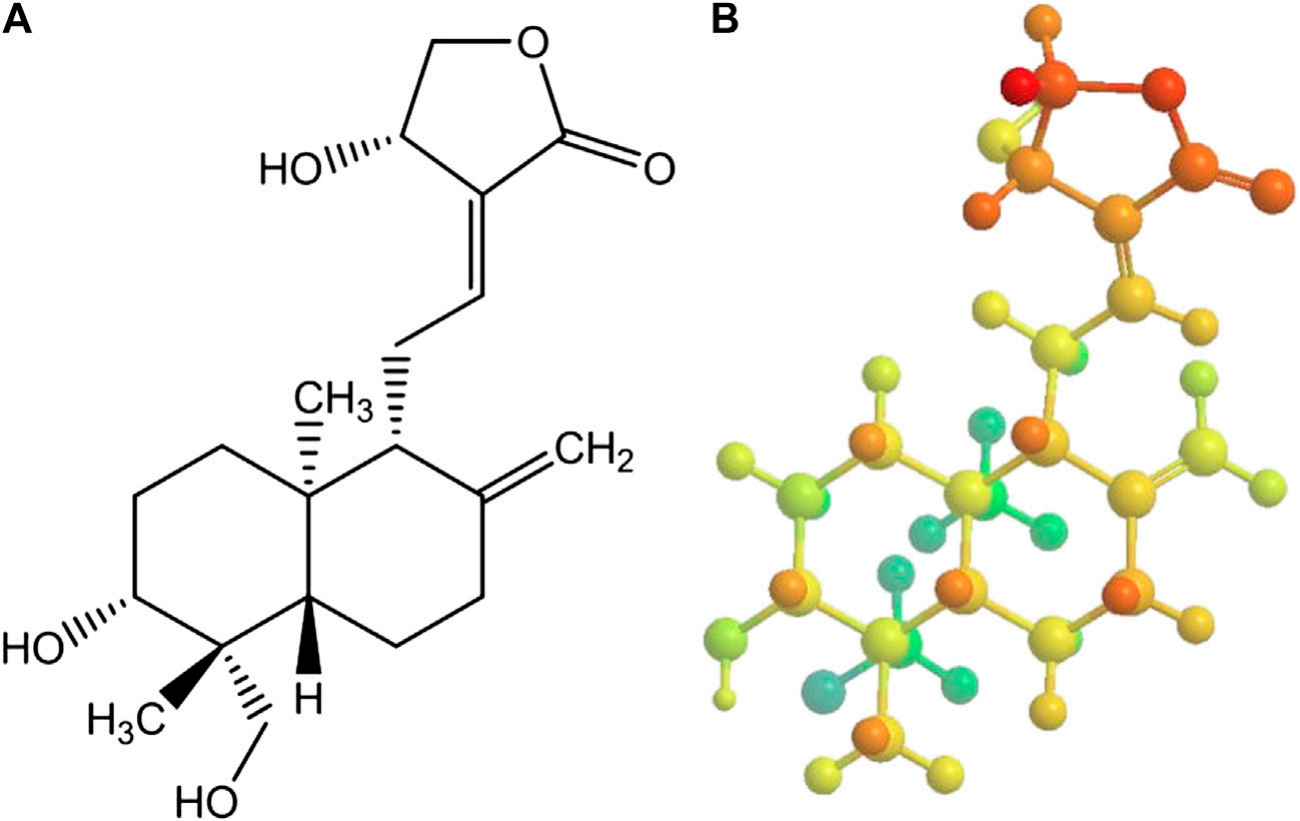
Structure of andrographolide **(A)**:2D and **(B)**:3D.

Enmozhi et al., 2020 has performed the evaluation of Andrographolide as a potential inhibitor of the main protease (Mpro) of SARS-COV-2 by using *in-silico* methods such as molecular docking. Andrographolide is a labdane type of diterpenoid which is isolated from *Andrographis paniculata* (Figure 10)*.* It was found to exhibit anti-HIV, anti-inflammatory and antineoplastic activities. But, molecular docking study was performed successfully on the binding site of SARS-CoV-2 Mpro. From the results of docking study, it was observed that negative values of free energy i. e −3.094357 kJ/mol suggest high affinity of Andrographolide for the binding site (Supplementary Table S5). This compound also follows drug-likeness rule i. e, Lipinski’s rule of five and makes it a promising drug molecule for further biochemical investigation to explore its potential utilization during treatment of COVID-19 (Gupta et al., 2017).

Currently, the clinical investigation reports implied that the anti-influenza drug like umifenovir is effective against COVID-19. Moreover, umifenovir intercalates mainly with the membrane lipids to inhibit the fusion of the viral particles with host membrane that blocks the entrance point of the virus inside the host cell. Similarly, another anti-influenza drug oseltamivir, which reduces the infection of the respiratory tract by blocking viral neuraminidase thereby, inhibits the viral particles from escaping host cells. So, it was found to be efficient for the treatment of COVID-19 infection. Oseltamivir, umifenovir is under phase III and phase IV clinical trial respectively (Saha et al., 2020).

## Future Perspective

Various pharmaceutical companies, research groups and laboratories are involved in the preparation of vaccines for the present as well as future epidemics incidence that utilize the broad-spectrum antibiotics (azithromycin), anti-viral drugs (remdesivir, favipiravir, lopinavir) or anti-malarial drugs (hydroxychloroquine) (Gennaro et al., 2020). So, drug repurposing study and clinical trials are extensively carried out for drug targeting approach. This study provides information regarding different drug targets from a structural part of virus and host cells in relation to the reported therapeutically active compounds with activity against SARS-CoV-2. Similarly, the results obtained from preclinical experiments reveal that PF-00835231 is recognized as a potent inhibitor of CoV-2 3CLpro with suitable pharmacokinetic (PK) properties that leads to further development of new drug candidates for the efficient treatment for COVID-19. For future perspective, there should be research collaboration that focus on target based approaches to fight against infections of SARS-CoV-2 and also evaluate the viral genetic structure in the future (Hsu et al., 2020; Seth et al., 2020).

## Conclusion

COVID-19 pandemic is not only affecting the public health but also make the entire globe in profound economic and psychosomatic distress. As, there is no suitable clinical management or vaccines available for COVID-19, research groups are extensively involved to identify the genome of the novel corona virus and utilize these concepts to develop new drug candidates against the different therapeutic targets of novel coronavirus (SARS-CoV-2). In this regard, some existing drugs have been repurposed to identify possible therapeutic agents that active against SARS-Cov-2. So, the most promising antiviral medications like remdesivir, favipiravir and antimalarial drugs such as chloroquine, hydroxychloroquine are evaluated clinically for safety and efficacious treatment of COVID-19. From clinical observations, it is reported that the antiviral drug like favipiravir prevents the replication phase of the virus life-cycle that leads to significant progress in clinical treatment of patients with mild to moderate infection with COVID-19. Similarly, remdesivir is the viral RNA-dependent RNA polymerase inhibitor. So, the drug repurposing study is emerged as a time-efficient and cost-effective strategy to determine new therapeutic indications for previously approved drug molecules.
